# Maximum tumor diameter and renal function can predict the declining surface dose rate after ^177^Lu-Dotatate: preliminary results of single institution in Japan

**DOI:** 10.1007/s11604-024-01585-5

**Published:** 2024-05-10

**Authors:** Takashi Ono, Mayumi Ichikawa, Takeo Tanada, Chika Kanezawa, Hiraku Sato

**Affiliations:** 1 Department of Radiation Oncology, Faculty of Medicine, Yamagata University, 2-2-2, Iida-Nishi, Yamagata, 990-9585 Japan; 2 Department of Radiology, Yamagata University Hospital, 2-2-2, Iida-Nishi, Yamagata, 990-9585 Japan

**Keywords:** Peptide receptor radionuclide therapy, ^177^Lu-Dotatate, Dose rate, Tumor diameter, Renal function

## Abstract

**Purpose:**

This study aimed to develop a user-friendly prediction formula for dose rate adjustment after initial ^177^Lu-Dotatate therapy from a prospective observational study of patients.

**Materials and methods:**

This study included consenting patients in a prospective observational study who underwent their first treatment in four cycles of ^177^Lu-Dotatate treatment at our hospital between January 2022 and February 2024. All patients received 7.4 GBq of ^177^Lu-Dotatate. The prediction formula was derived from the regression analysis of tumor-related factors and renal function. Creatinine clearance was estimated using the Cockcroft–Gault equation in this study for renal function.

**Results:**

Among the 13 patients (seven males, six females, median age: 59 years), logarithmically transformed total tumor volume (cc) and maximum tumor diameter (mm) of primary tumors or metastases showed strong correlations (*p* < 0.001, R^2^ = 0.897). As such, the maximum tumor diameter was used as the tumor parameter in the prediction formula. Additionally, maximum tumor diameter and creatinine clearance showed strong (*p* < 0.001, R^2^ = 0.768) and moderate (*p* = 0.013, R^2^ = 445) correlations, respectively, with the ratio of the dose rate 5.5-h post-administration to the dose rate immediately post-administration (%) at 1 m from the body surface. The resulting formula, 51.4 + 0.360 × maximum tumor diameter (mm) − 0.212 × creatinine clearance (ml/min), demonstrated an extremely strong correlation (*p* < 0.001, R^2^ = 0.937).

**Conclusion:**

The present study showed that the maximum tumor diameter and renal function affected the declining the dose rate of patients surface after ^177^Lu-Dotatate, which can inform post-administration dose rate management and potentially facilitate outpatient treatment in Japan.

## Introduction

Neuroendocrine neoplasms (NENs) are relatively rare tumors that can be distributed in almost all anatomical sites and organs in the body. These tumors are further classified into neuroendocrine tumors (NETs) or neuroendocrine cancers (NEC). NETs are well-differentiated neoplasms with potential to metastasize or invade adjacent organs, whereas NECs often exhibit malignant behavior. Furthermore, NETs are graded (grades 1–3) based on their mitotic count and Ki-67 labeling index [1].

According to Surveillance, Epidemiology, and End Stage, the incidence of NETs, including gastroenteropancreatic (GEP) NETs, has gradually increased over the past 40 years possibly due to improved detection methods and earlier stage diagnoses [2, 3]. In Japan, a total of 6735 patients were treated for GEP-NENs in 2016, resulting in an age-adjusted overall incidence of 3.53 per 1000,000 population. Notably, approximately 80% of duodenal, appendiceal, and rectal NENs in this population were classified as grade 1. Surgical or endoscopic resection remains the preferred treatment for most GEP-NENs, with the exception of esophageal NENs [4].

However, not all patients are eligible for surgery due to advanced-stage disease with adjacent organ invasion or distant metastasis. Such cases are treated with systemic therapy, including somatostatin analogs, molecular targeted therapy, and cytotoxic agents. Several studies have also explored other optimal treatment methods based on liver metastasis tumor volume, Ki-67 labeling index, overall tumor volume, and growth rate [5, 6].

New treatment methods have recently emerged in the phase 3 NETTER-1 trial. Particularly, peptide receptor radionuclide therapy using ^177^Lu-Dotatate showed markedly longer progression-free survival and a significantly higher response rate compared to long-acting octreotide monotherapy in patients with grade 1 or 2 midgut NETs [7]. Although, there was no significant median overall survival time after long-term follow-up due to crossover treatment [8]. Moreover, ^177^Lu-Dotatate demonstrated remarkable tumor shrinkage and tolerability with no severe toxicities in Japan [9]. Studies have even suggested potential positive effects in other tumor locations beyond the midgut, although results vary depending on the primary site [10, 11]. Typically, ^177^Lu-Dotatate is administered in four cycles, and some reports observed that patients who received higher cumulative doses (29.6 GBq) showed better response and survival [12]. In addition, the NETTER-2 trial has provided new evidence supporting the use of ^177^Lu-Dotatate as first-line therapy, demonstrating significantly improved progression-free survival compared to long-acting octreotide monotherapy for grade 2 or 3 midgut NETs [13].

In September 2021, Japan approved ^177^Lu-Dotatate administration for national insurance coverage. However, there is limited infrastructure to accommodate this therapy, with only 66 nuclear medicine rooms and 160 beds as of 2022 [14]. Consequently, many patients have been placed on waiting lists, and resource shortage is expected with policy changes based on promising results from the NETTER-2 trial [13]. As such, effective management of available resources is crucial to address these limitations. In Japan, patients can be discharged after ^177^Lu-Dodtate therapy if the measured dose rate at 1 m from the body surface falls below 18 μSv/h [15]. For this reason, it is important for management to predict how quickly they can leave. However, there are no data about it. However, relevant data on this matter are lacking. Therefore, the purpose of this study was to derive a user-friendly prediction formula for decreasing dose rate after ^177^Lu-Dotatate administration based on collected data from a prospective observational study.

## Materials and methods

### Ethics statement

This prospective study was approved by the Institutional Ethics Committee of the Faculty of Medicine at Yamagata University (approval number: 2021-297). The study was conducted in accordance with the principles of the Declaration of Helsinki.

### Patients

This study included consenting patients in a prospective observational study who underwent their first four cycles of ^177^Lu-Dotatate treatment at our hospital. A total of 14 patients with NETs received ^177^Lu-Dotatate treatment at our hospital between January 2022 and February 2024; however, one patient was excluded due to disagreement with the prospective observational study. Data analysis was limited to the first treatment cycle. The reason why we limited the data from cases that received the first treatment in four cycles was because there was one patient that the dose rate reduction rate significantly increased after subsequent cycles despite no significant changes in blood sampling data.

### Treatment methods

Patients were evaluated by a multidisciplinary team to determine eligibility for ^177^Lu-Dotatate treatment. The inclusion criteria were as follows: (1) pathologically confirmed NETs, (2) grade 2 or higher uptake (grade 2: equivalent to normal liver tissue; grade 3: greater than normal liver tissue, grade 4: higher than normal spleen or kidney tissue) by ^111^In pentetreotide scintigraphy [16], (3) platelet count ≥ 75,000 /μl, (4) creatinine clearance ≥ 40 ml/min, and (5) having no adverse events equal to or exceeding grade 3 classification based on the Common Terminology Criteria for Adverse Events version 5.0 [17]. Creatinine clearance was estimated using the Cockcroft–Gault equation in this study [18], which was consistent with the NETTER-1 trial [7, 8]. The equation considers the patient’s age in years, actual body weight in kg, and plasma creatinine level (Pcr). Final creatinine values for female subjects are reduced by multiplying the initial result by 0.85. The equation is presented below:

$$\frac{\left(140-{\text{Age}}\right)\times \mathrm{Body weight}}{72\times {\text{Pcr}}}.$$

After creatinine clearance estimation, patients received a 5HT_3_ receptor antagonist 30 min before treatment, followed by 1000 ml of l-lysine/l-arginine hydrochloride 4 h later. Afterward, 7.4 GBq of ^177^Lu-Dotatate is administered 30 min after initiating l-lysine/l-arginine hydrochloride administration and was completed within 30 min. Dose rate measurements at 1 m from the patient’s body surface were performed immediately after ^177^Lu-Dotatate treatment and 5.5-h post-administration using an ICS-311 device (ALOKA, Tokyo, Japan). Patients were asked to urinate 10 min before the start of ^177^Lu-Dotatate administration, and then were asked to refrain from urinating until dose rate measurements immediately post-administration. No restrictions or adjustments were made for urination at the time of 5.5-h measurement.

### Statistics

As most of the excretion process of ^177^Lu-Dotatate occurs through the kidneys, and treatment is performed for cases with high accumulation in tumors [18], it is hypothesized that total tumor volume and renal function would affect drug excretion. However, all patients had an uptake score of 4, except for three cases with a score of 3. Thus, the degree of uptake could not be used for analysis due to limited variation. The total tumor volume was calculated by contouring all volumes of primary and metastatic tumors using MIM Maestro version 7.2.8 (MIM, Ohio, USA), although its calculation is often difficult in clinical practice. To address this, regression analysis was performed to examine the correlation between tumor diameter and total tumor volume for all primary and metastatic lesions, because it was predicted that the larger the maximum tumor diameter, the larger the total tumor volume. For renal function, creatinine clearance was used for its evaluation. After calculating both parameters, regression analysis was performed to investigate the correlation between tumor parameters or creatinine clearance and the ratio of the dose rate 5.5-h post-administration to the rate immediately post-administration (%) at 1 m from the patient’s body surface. Statistical analyses were performed using the IBM SPSS Statistics software (version 24; SPSS Inc., Chicago, IL, USA), and all *p*-values were two sided, with statistical significance set at *p* < 0.05.

## Results

### Patient

Patient characteristics are summarized in Table 1. The study included seven males and six females, with a median age at treatment of 59 years. The median total tumor volume and maximum tumor diameter were 116.5 cc and 50.3 mm, respectively. The median creatinine clearance was 74.1 ml/min (range: 53.5–139.5 ml/min). Doses of ^177^Lu-Dotatate were not reduced for any patient, and all patients were treated as scheduled. The median dose rate immediately post-administration was 41.0 μSv/h (range: 35.0–46.0 μSv/h).

**Table 1:** Patient characteristics

Patient	Age	Gender	Performance status	Primary site	Pathology NET grade	Metastases site	Tumor volume (cc)	Maximum diameter of tumor (mm)	Creatinine clearance (ml/min)
1	64	Male	0	Rectum	2	Liver, bone, lymph node	1958.6	85.9	58.5
2	55	Female	0	Pancreas	1	Liver, lymph node	25.5	24.6	104.4
3	59	Female	1	Pancreas	3	Liver, bone	275.9	80.4	53.5
4	45	Male	0	Rectum	2	Liver, bone, lymph node	356.9	60.4	104.6
5	57	Male	0	Pancreas	2	Liver, lymph node	10.0	18.5	65.7
6	72	Male	0	Lung	2	Liver, lymph node	2538.6	96.5	60.7
7	64	Female	0	Pancreas	3	Liver, lymph node	79.0	27.8	74.1
8	67	Female	0	Rectum	2	Liver, bone, lymph node, subcutaneous	166.8	50.3	81.0
9	61	Male	0	Duodenum	2	Liver	26.3	29.0	104.2
10	57	Male	0	Rectum	2	Liver, lymph node	67.2	49.6	56.9
11	52	Female	0	Pancreas	2	Liver, lymph node, kidney	17.7	19.5	81.6
12	39	Male	0	Pancreas	2	Liver, lymph node	116.5	58.1	139.5
13	77	Female	0	Retroperitoneum	2	Liver	238.4	61.0	59.3

*NET* neuroendocrine tumor

### Correlation between maximum tumor diameter and total tumor volume

**Fig. 1:**
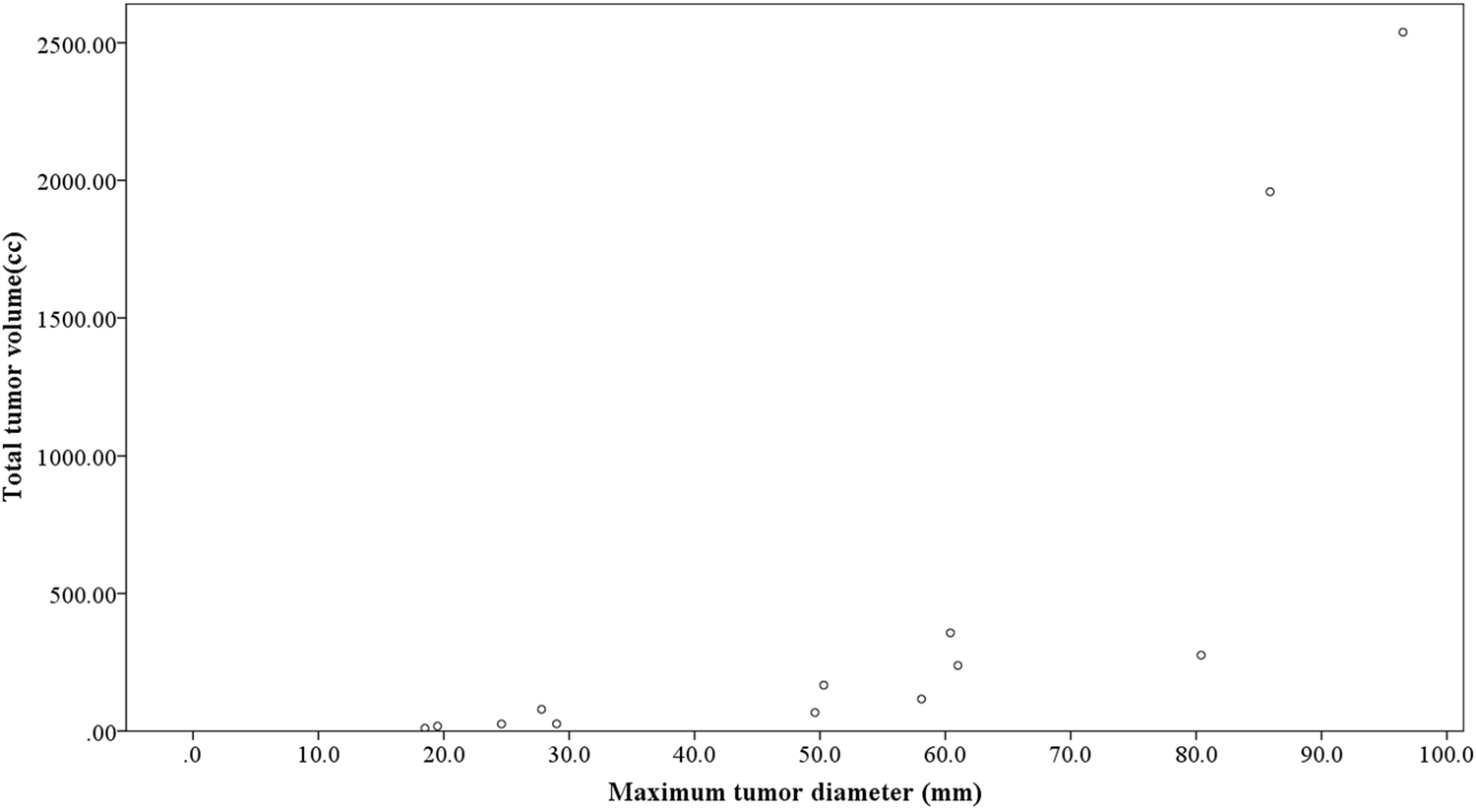
The correlation between maximum tumor diameter and total tumor volume

**Fig. 2:**
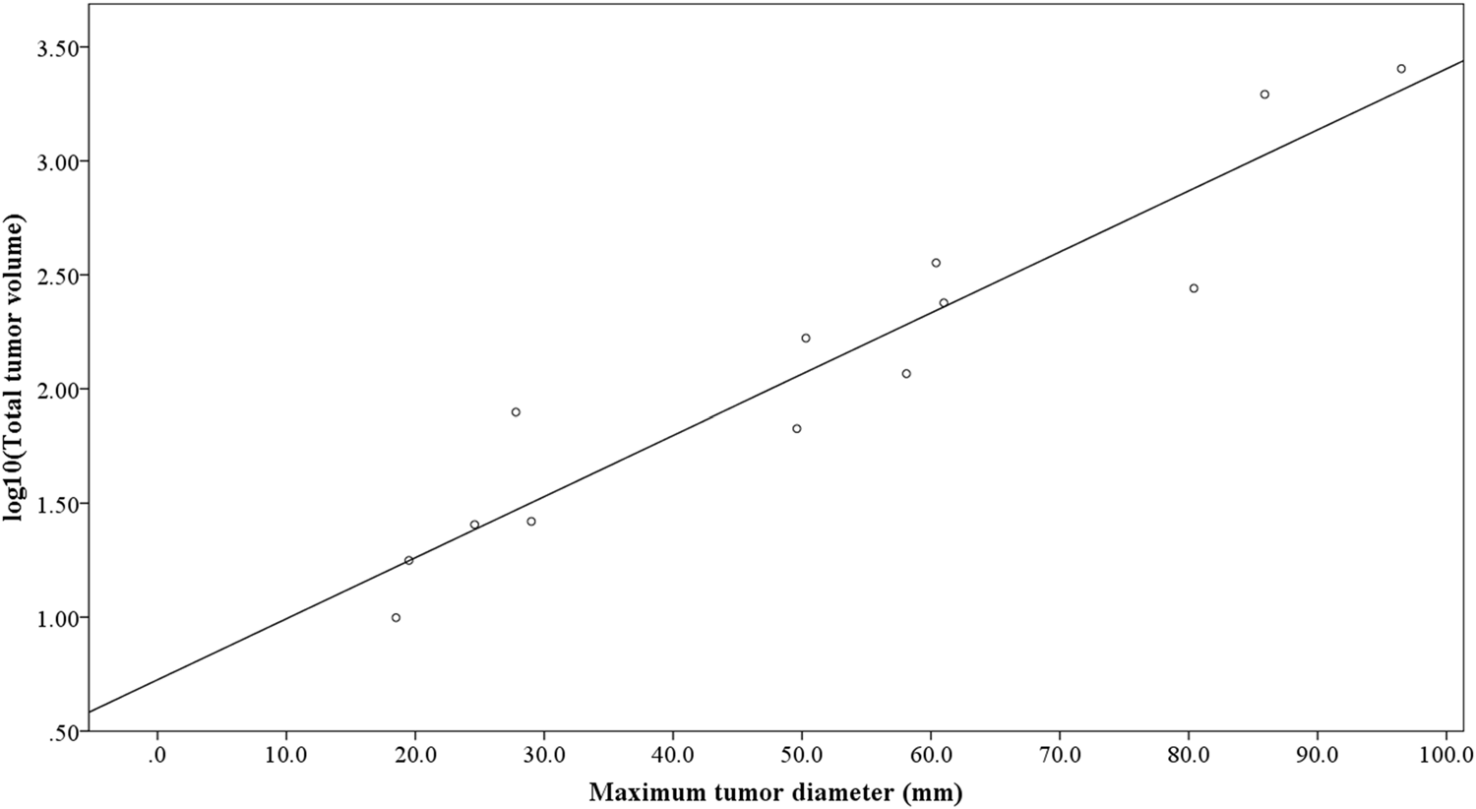
The correlation between maximum tumor diameter and logarithmically transformed numbers total tumor volume

A scatter plot illustrating the relationship between maximum tumor diameter and total tumor volume is presented in Fig. 1. Due to the possibility of an exponential correlation, total tumor volume data underwent logarithmic transformation. Subsequent regression analysis revealed a strong correlation between the two parameters (*p* < 0.001, R^2^ = 0.897) (Fig. 2). Based on this finding, maximum tumor diameter was chosen as the tumor-related parameter for further analysis.

### Regression analysis for dose rate reduction

**Fig. 3:**
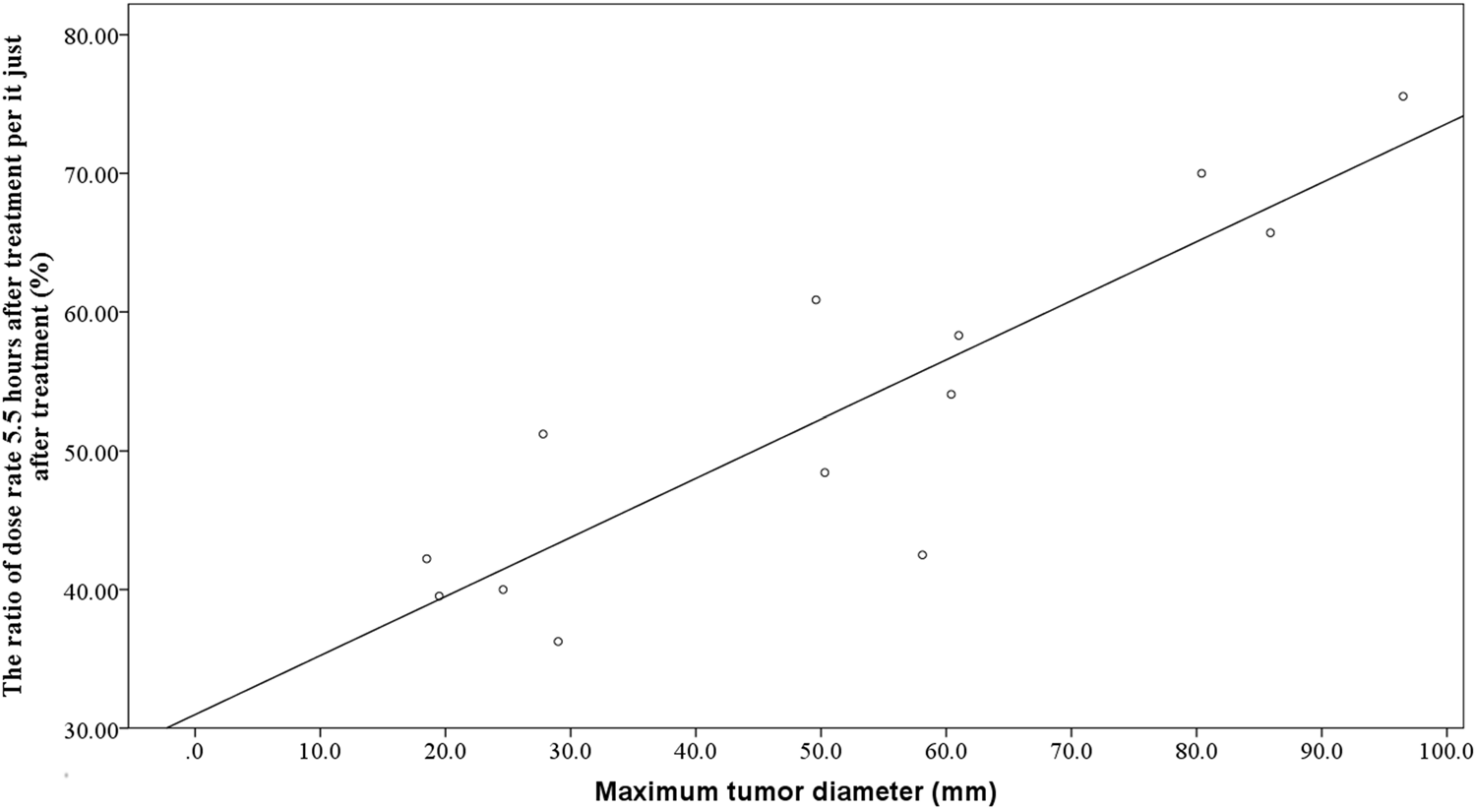
The correlation between maximum tumor diameter and the ratio of dose rate 5.5 h after treatment per it just after treatment

**Fig. 4:**
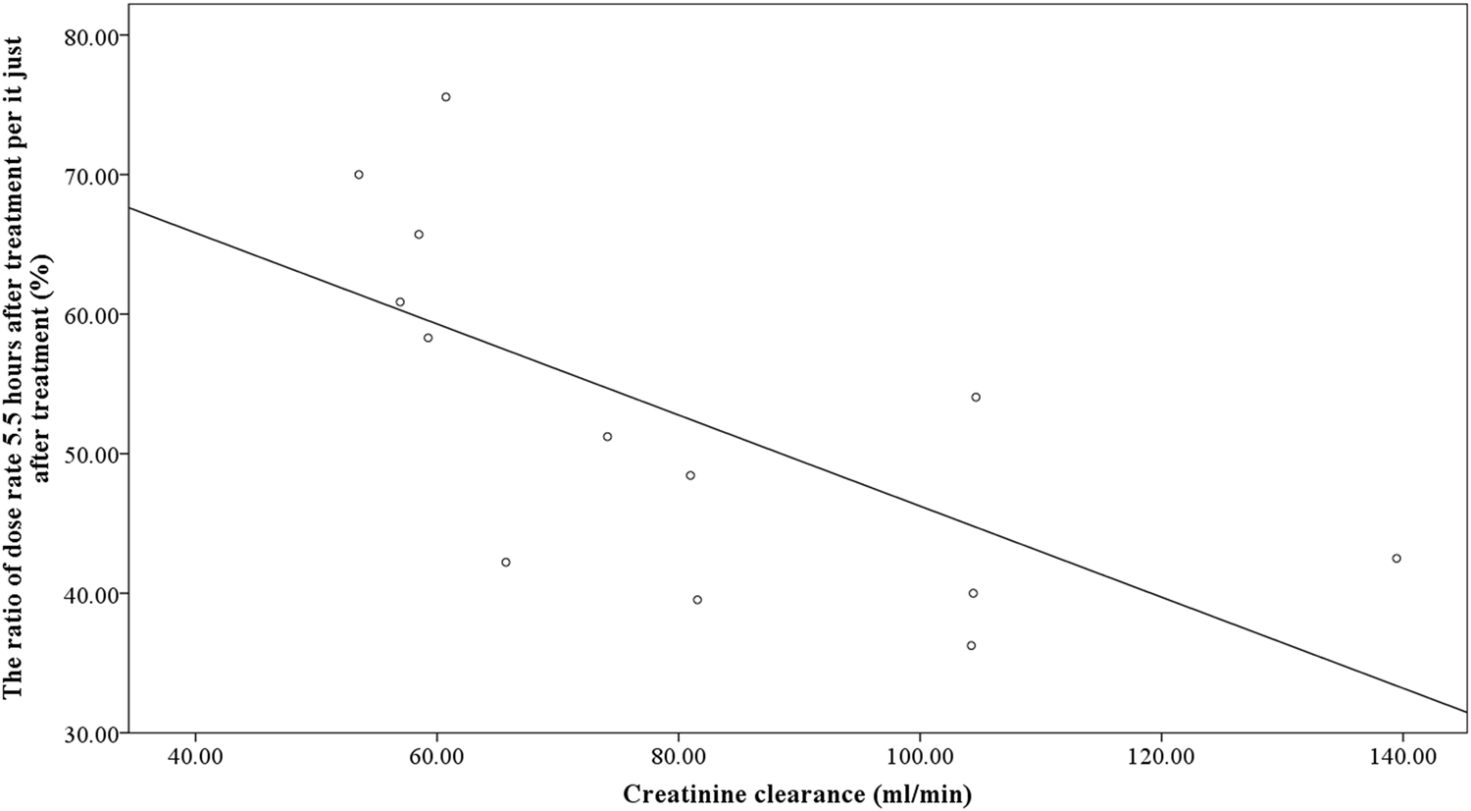
The correlation between creatinine clearance and the ratio of dose rate 5.5 h after treatment per it just after treatment

A scatter diagram illustrating the relationship between the maximum tumor diameter and dose rate ratio is shown in Fig. 3. Regression analysis revealed a strong correlation (*p* < 0.001; R^2^ = 0.768) between the two. Similarly, a scatter diagram demonstrating the relationship between creatinine clearance and dose rate ratio is shown in Fig. 4. Regression analysis of these two parameters revealed a moderate correlation (*p* = 0.013, R^2^ = 445). To develop a prediction formula for the dose rate ratio, multivariate regression analysis was performed using the maximum tumor diameter and creatinine clearance. The analysis revealed an extremely strong correlation between the two parameters (*p* < 0.001, R^2^ = 0.937), with standardized partial regression coefficients of 0.739 and − 0.434 for maximum tumor diameter and creatinine clearance, respectively. Additionally, the partial regression coefficient was 0.360 (*p* < 0.001, 95% confidence intervals = 0.269–0.450) for maximum tumor diameter, and − 0.212 (*p* < 0.001, 95% confidence intervals = − 0.303 to − 0.121) for creatinine clearance, respectively. Based on these results, the following prediction formula was developed: 51.4 + 0.360 × maximum tumor diameter − 0.212 × creatinine clearance. The error of the predicted value using this formula among the patients of present study was − 5.5 to 4.2%.

## Discussion

To the best of our knowledge, this is the first study to present a prediction formula for the dose rate ratio in patients with NETs after undergoing initial ^177^Lu-Dotatate administration.

Our findings showed that the largest diameter of all tumors, including metastases, was exponentially correlated with total tumor volume. Appropriate calculation and evaluation of total tumor burden is extremely complicated and impractical in actual clinical practice, especially for very extensive and advanced cases. Considering that ^177^Lu-Dotatate is used in unresectable cancers, total tumor burden calculation becomes impractical. As such, measuring the maximum tumor diameter presents a simpler and more feasible approach.

Statistical analysis of the present study showed that the maximum tumor diameter (total tumor volume) served as a better predictor of dose rate decline compared to creatinine clearance (renal function). Since ^177^Lu-Dotatate treatment targets tumors exhibiting high uptake on octreotide scintigraphy, increased uptake, longer retention in the tumor, and delayed excretion from the body are expected in large tumor masses. Furthermore, this implies that exceeding a certain tumor size could hinder dose rate reduction and prolong fulfillment of discharge criteria, regardless of renal function.

Water intake is another factor that can affect dose rate reduction, as previously reported by Ni et al. [20] who observed lower dose rates in patients with 24-h water consumption of no less than 2750 ml. This effect may be due to the impact of water intake on renal function. Similarly, Kim et al. [21] reported that among acyclovir-treated patients, renal function indicators were within normal limits in those with a hydration volume > 2 L/day compared to patients with a hydration volume < 2 L/day. These results suggest that the addition of oral or intravenous fluid replenishment may promote renal excretion and expedite dose rate decline. Although patients in our study had uniform intravenous dosages despite non-standardized oral fluid intake, we believe that the influence on the results was minimal. This is because renal function is typically lower compared to tumor-derived parameters, and because the time spent drinking water was relatively short (less than a quarter of the day).

The potential to predict dose rate reductions offers significant clinical advantage beyond room management. Its success could also facilitate the exploration of outpatient treatment options. Based on our study’s estimated intermediate dose rate of 41.0 μSv/h at treatment completion, achieving the standard exit criteria of 18 μSv/h (43.9% the immediate dose rate) within 5.5 h would require varying levels of creatinine clearance based on tumor diameter. Specifically, for tumor diameters of 10, 20, 30, 40, and 50 mm, creatinine clearances of 52.4, 69.4, 86.4, 103.4, and 120.4 ml/min are required, respectively. On the other hand, assuming a maximum observed dose rate of 46.0 μSv/h in the present study, creatinine clearances of 75.9, 92.4, 108.9, 123.4, and 141.9 ml/min are required to achieve the same result. In fact, there was one patient with maximum tumor diameter of 29 mm received the third and fourth treatments on outpatient basis for three and four cycles, respectively, with a creatinine clearance of 104.2 ml/min, which fell within the predicted range. Although it is not possible to completely rule out the possibility that renal function decline may occur with subsequent administration, no significant decline in renal function was reported in the NETTER-1 trial [7], so we believe that this possibility is low. Although it is necessary to evaluate renal function through blood sampling until the next administration, we believe that it is reasonable to make predictions based on renal function evaluation at the time of the first administration. Thus, the ability to determine whether the patient will be able to undergo outpatient treatment would streamline treatment scheduling and improve resource allocation and management. Furthermore, as suggested by Ni et al. [20], additional fluid intake of approximately 500 ml within 2 h after treatment might offer further dose rate reduction.

Despite the valuable insights offered by this study, three limitations must be acknowledged. First, given the single-institution design of the study, the sample size was relatively small and heterogeneous. However, our findings of a strong and significant correlation between the study parameters remains impactful despite the limited sample. Second, the study did not account for the influence of octreotide scintigraphy uptake score. However, due to minimal variations in uptake scores and performance of octreotide scintigraphy at other facilities in some patients, a comprehensive evaluation was deemed infeasible. Nevertheless, we believe that the development of a user-friendly prediction formula holds significant value in clinical practice. Third, present study is based on one point in time, 5.5 h later, and it is completely impossible to predict how dose rate will change before and after that point. However, if we can predict whether the decline will occur by the evening we can predict whether or not it is possible to administer it as an outpatient even at one point. Therefore, we think it has some meaning. In the future, it is expected that detailed analysis using larger number of patients and analysis using multiple SPECT/CT measurements for dosimetry with artificial intelligence will better predict declining trends of surface dose rate to justify the proposed formula in the present study.

In conclusion, the present study showed that the maximum tumor diameter and renal function affected the declining the dose rate of patients surface after ^177^Lu -Dotatate administration. The derived prediction formula was as follows: ratio of dose rate 5.5-h post-administration to the dose rate immediately after treatment (%) = 51.4 + 0.360 × maximum tumor diameter − 0.212 × creatinine clearance. Considering that the number of target cases will increase in the future, as reported in the NETTER2 trial [13], this formula offers potential utility in the management of treatment rooms and facilitation of outpatient treatments in Japan.
